# Correspondence

**Published:** 1872-11

**Authors:** E. M. Vasser

**Affiliations:** Cahaba, Dallas Co., Ala.


### CORRESPONDENCE.

CAHABA, DALLAS CO., ALA., August 10th, 1872.

*Editors Companion, Atlanta, Georgia.*

GENTLEMEN:—Thinking you would like to hear from this part of the world, I herewith submit to the pages of your valuable journal a report of a case that came under my observation in the city of Mobile. On the 15th of June last, I had the pleasure of an invitation from Dr. J. T. Gilmore, Alabama's gifted surgeon, to visit a Spaniard, aged 67 years, on the corner of Bayon near Conti streets, and witness the removal of the tongue.

The operation was performed with Dr. Nott's Rectilineal Ecraseur. An incision was made through the centre of the lower lip and extended to the hyoid bone, and then with a chain saw divided the inferior maxilla through the symphasis, then detached the tongue from the lower jaw and applied Dr. Nott's Rectilineal Ecraseur, by the aid of which it was removed as far back as the palatine and without the loss of blood, the patient being anæsthetized during the entire operation, which was performed for cancer of the tongue, deeply seated in the muscular structure of the organ, the disease extending from the attachment of the organ to the lower jaw back to its dorsum. After the Ecraseur was removed I looked for hemorrhage, as this is one of the bloodiest of operations, but there was none. No use for a ligature. The necessary bandages were applied, etc. After fully reacting from the anæsthetic, he conversed freely with his friends and family, who were as much surprised as myself to hear him talk; they asked him many questions, and his answers were interpreted, but as we did not understand the Spanish language sufficiently to carry on a conversation, we cannot say how good his articulation was. A small portion of morphæ sulph. was ordered. Food directed to be administered by means of a tube, and as we were about to retire the poor fellow grasped the surgeon by the hand with both of his, and from his continued talk, emphesis and expression of every feature, I felt satisfied he was trying to manifest his gratitude to his benefactor.

On our arrival at the office another case, if I recollect, a Mobilian, aged fifty-five years, presented himself for examination. He had had half of his tongue cut off just two (2) weeks before for the same cause; the wound had healed entirely. I had considerable conversation with him. He had an impediment, however, which showed some defect in the organs of speech, though no one would suppose for one moment, he had lost half of his tongue. On the 19th of June Dr. G. invited me to take a seat in his buggy and see the Spaniard. I did so, found the wound healing by the first intention, and the temporal pain from which he had suffered so long and severely, was now relieved, and although he had taken severe cold, I think the chances are all in his favor for a speedy recovery.

Another patient presented himself in May, to pay his respects to Dr. G., who had his entire tongue taken off two years ago. Since writing the above I learn the Spaniard has recovered. The other case, aged 55, died several days since, with pneumonia. The Ecraseur of Dr. Nott deserves special mention. It is simple enough to look at, yet it took his brain to conceive the idea of the formation of such an instrument; he has certainly done much to alleviate suffering humanity. Dr. Gilmore succeeds him in Mobile. He is a middle aged man, a courteous and dignified gentleman; a thorough anatomist; a surgeon; a fearless, yet cautious operator.

Hoping I have not trespassed too much on your space, and with many wishes for the success of your valuable journal, I remain, gentlemen, very truly, yours,

E. M. VASSER, M. D.

---

